# Supplementary material for: The Diversity, Resistance Profiles and Plasmid Content of Klebsiella spp. Recovered from Dairy Farms Located around Three Cities in Pakistan
Source: Antibiotics (Basel). 2023 Mar 8;12(3):539. doi: 10.3390/antibiotics12030539 (PMC10043998; doi:10.3390/antibiotics12030539)

Supplementary Table S1. Serotypes and sequence types of sequenced *Klebsiella* spp. isolates.

Strain	City	Farm	ST	Wzi	K_locus	O_locus
P500	Quetta	B	ST11	wzi150	unknown	OL101
P501	Quetta	A	ST985	wzi39	KL39	O1v2
P502	Quetta	T	ST1446	wzi39	KL39	O3b
P503	Quetta	T	ST37-1LV	wzi14	KL14	O3b
P505	Quetta	T	ST3449	wzi507	KL14	O5
P506	Quetta	T	ST687-1LV	wzi39	KL39	O3/O3a
P507	Quetta	T	ST105-1LV	wzi459	KL20	O3b
P508	Quetta	T	ST63-2LV	wzi23	KL14	O3/O3a
P509	Quetta	Al-K	ST4075	wzi150	unknown	OL101
P510	Quetta	Al-K	ST1109	wzi115	KL54	O1v2
P511	Quetta	Al-K	ST736	wzi171	KL53	O3/O3a
P512	Quetta	Al-K	ST4461-1LV	wzi86	KL125	O5
P513	Quetta	Ak	ST2390	wzi105	KL103	O1v1
P514	Quetta	Ak	ST218-1LV	wzi77	KL57	O2v2
P515	Quetta	Ak	ST1037	-	KL27	O2v2
P516	Quetta	Ak	ST37	wzi14	KL14	O3b
P517	Quetta	Bi	ST2055	wzi386	unknown	OL104
P518	Quetta	Bi	ST104	wzi102	KL31	O2v2
P519	Quetta	Bi	ST2320-2LV	wzi504	KL1	O3/O3a
P520	Quetta	Bi	ST3067	wzi324	KL63	O3b
P521	Quetta	Bi	ST2118	wzi417	KL46	O3b
P522	Quetta	Bi	ST1731	wzi162	KL122	O1v2
P523	Quetta	Bi	ST37	wzi14	KL14	O3b
P524	Quetta	Sa	ST1833-1LV	wzi64	KL64	O2v1
P525	Quetta	Sa	NA	-	unknown	unknown
P526	Quetta	Sa	ST3186	wzi19	KL19	O2v2
P527	Quetta	Sa	ST37	wzi14	KL14	O3b
P529	Quetta	Sa	NA	-	unknown	unknown
P530	Quetta	Sa	ST46	wzi64	KL64	O2v1
P537	Sargodha	U	ST995	wzi29	KL106	O1v2
P538	Sargodha	U	ST11	wzi150	unknown	OL101
P540	Sargodha	U	ST1391-1LV	wzi151	KL48	O1v1
P541	Faisalabad	SH	ST1315	wzi432	KL64	O2v2
P542	Faisalabad	SH	ST605	wzi42	KL42	O4
P543	Faisalabad	SH	ST2219-1LV	wzi447	unknown	OL101
P544	Faisalabad	SH	ST2187	-	unknown	O4
P545	Faisalabad	SH	ST1377-3LV	-	KL144	O4
P547	Faisalabad	SH	ST190-2LV	wzi50	KL17	O4
P548	Faisalabad	SH	ST355	wzi81	KL131	O5
P549	Faisalabad	SH	ST37-2LV	-	unknown	unknown
P550	Faisalabad	SH	ST1315	wzi432	KL64	O2v2
P551	Faisalabad	SH	ST1835	-	unknown	O3b
P552	Faisalabad	SH	ST45	-	KL153	O2v1
P553	Faisalabad	SH	ST17-1LV	wzi82	KL23	O2v2
P554	Faisalabad	SH	ST705-3LV	-	KL30	O12
P555	Faisalabad	SH	ST2876-1LV	wzi197	KL141	O4
P557	Faisalabad	SH	ST1708-1LV	-	unknown	OL103
P558	Faisalabad	SH	ST34-2LV	wzi81	KL120	O1v2
P559	Faisalabad	L	ST191	wzi42	KL42	O4

P565	Faisalabad	L	ST1412-1LV	wzi173	KL102	O2v2
P568	Faisalabad	L	ST37	wzi14	KL14	O3b

Supplementary Table S2. Kleborate mean resistance and virulence scores of Kp isolates from the current study compared to a global dataset.

	Pak-animal			Kleborate global dataset		
K. pneumoniae	Total	mean_vir	mean_res	total	mean_vir	mean_res
ST1037	1	0	0	6	0.166666667	0.166666667
ST104	1	0	0	9	1	1.222222222
ST105-1LV	1	0	0	1	1	0
ST11	2	1	1	1169	1.485885372	1.843455945
ST1109	1	0	0	6	0	0.5
ST1315	2	0	0	2	0	0
ST1377-3LV	1	0	0	N/A	N/A	N/A
ST1391-1LV	1	0	1	N/A	N/A	N/A
ST1412-1LV	1	0	0	N/A	N/A	N/A
ST1446	1	0	0	1	0	0
ST17-1LV	1	0	0	6	0	0.5
ST1731	1	0	0	3	0	0.666666667
ST1833-1LV	1	0	0	1	0	2
ST1835	1	0	0	N/A	N/A	N/A
ST190-2LV	1	0	0	N/A	N/A	N/A
ST191	1	0	0	1	0	0
ST2055	1	0	0	1	0	0
ST2118	1	0	0	2	0	1
ST218-1LV	1	0	0	N/A	N/A	N/A
ST2187	1	0	0	N/A	N/A	N/A
ST2219-1LV	1	0	0	N/A	N/A	N/A
ST2390	1	0	0	2	0	0.5
ST2876-1LV	1	1	0	N/A	N/A	N/A
ST3067	1	0	0	1	1	0
ST3186	1	0	0	1	0	0
ST34-2LV	1	0	0	2	2	0
ST37	4	0	0	200	0.325	0.73
ST37-1LV	1	0	0	9	0.333333333	1.333333333
ST37-2LV	1	0	0	1	0	3
ST4075	1	0	0	N/A	N/A	N/A
ST4461-1LV	1	0	0	N/A	N/A	N/A
ST45	1	1	0	190	1.021052632	0.915789474
ST46	1	0	0	1	0	1
ST605	1	0	0	3	0	0
ST63-2LV	1	0	0	N/A	N/A	N/A
ST687-1LV	1	0	0	N/A	N/A	N/A
ST985	1	1	1	17	0.705882353	0.882352941
ST995	1	0	1	3	0	1

Supplementary Figure S1. Species diversity and distribution in cattle from 10 different dairy farms in **the cities of** Quetta, Faisalabad and Sargodha in Pakistan.

For the format used in this figure: https://microreact.org/project/gYYDfWDgs2eB7KAA7URKwW-species-distribution.

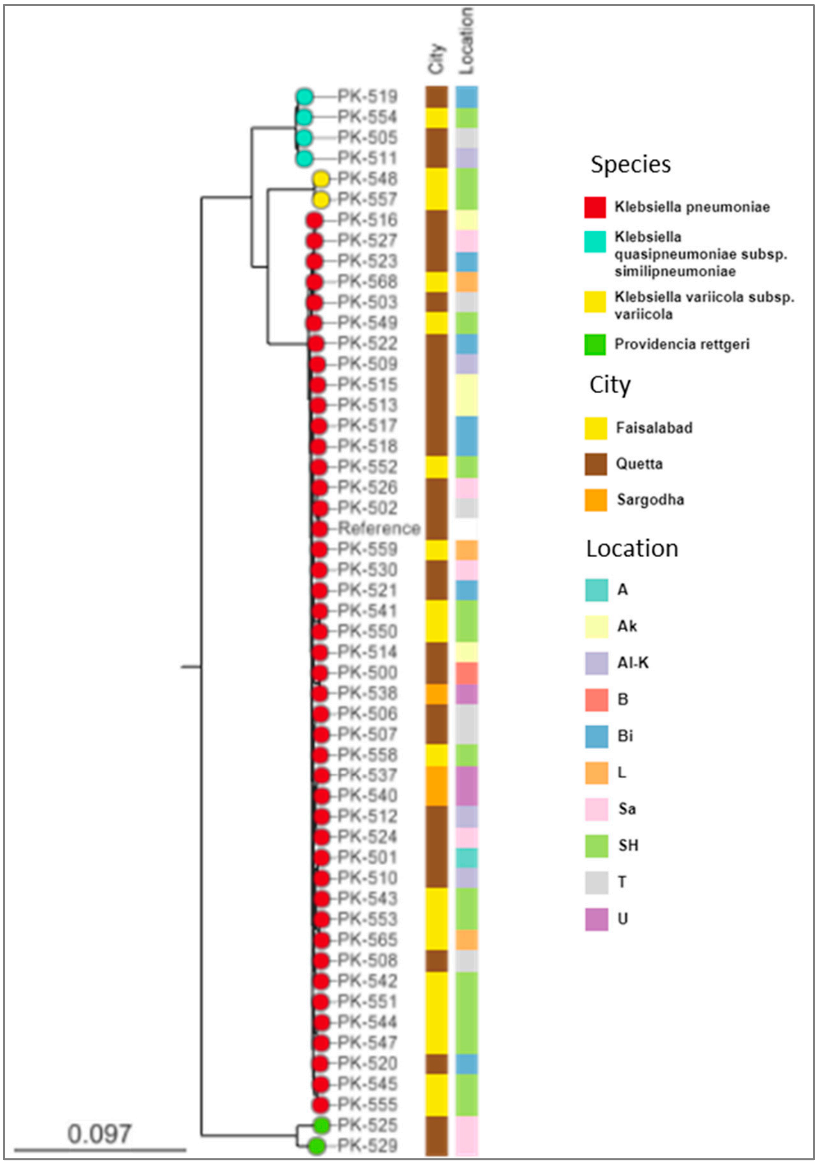

Supplementary Figure S2. AMR distribution among species.

https://microreact.org/project/c7qYSGsQg5dxgDjYXfPitN-amr-genes.

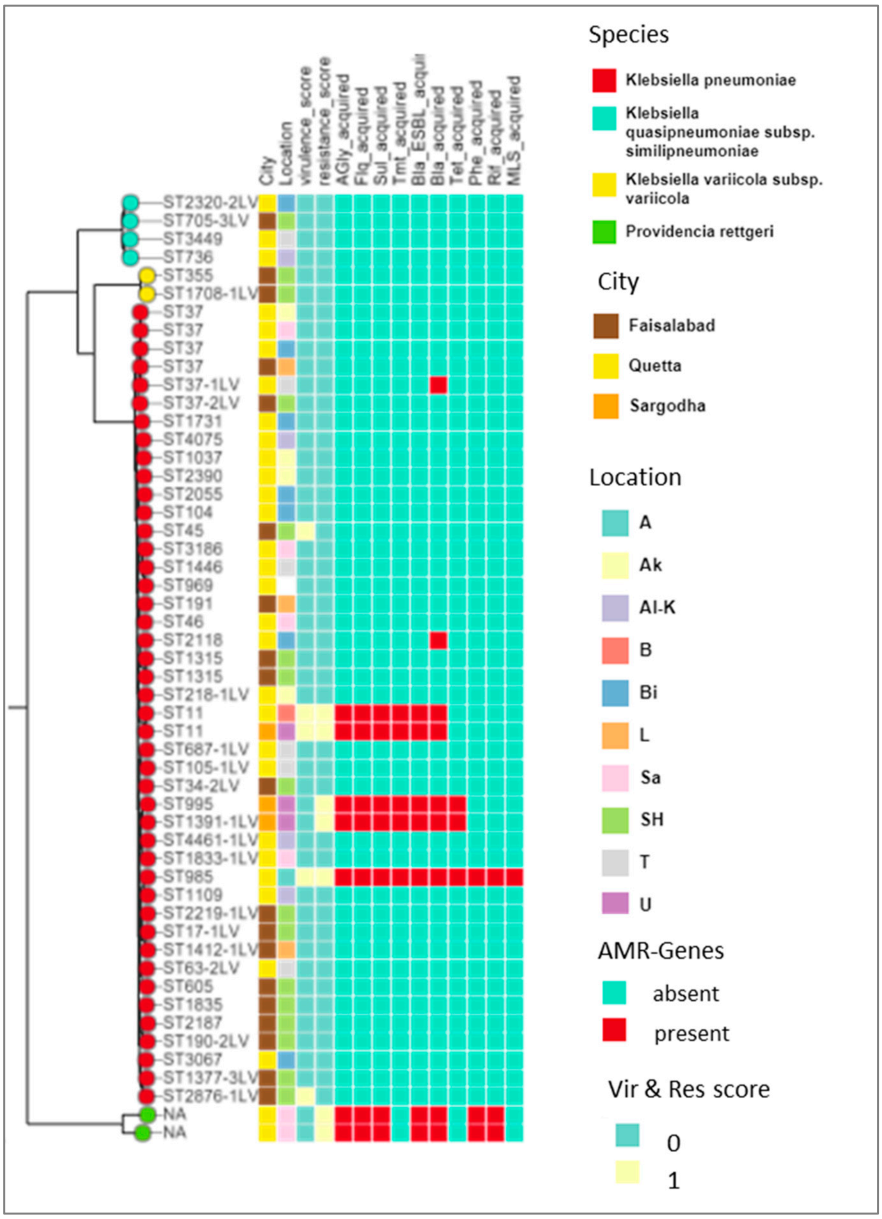

Supplementary Figure S3. Approximate maximum likelihood phylogenetic tree (midpoint rooted) of the 51 isolates analysed. Species and location of each isolate and presence of plasmids identified using MOB-suite are indicated. A red asterisk indicates plasmids associated with resistance genes.

https://microreact.org/project/eJFkQuN5YKyTagqK8FG44P-plasmid.

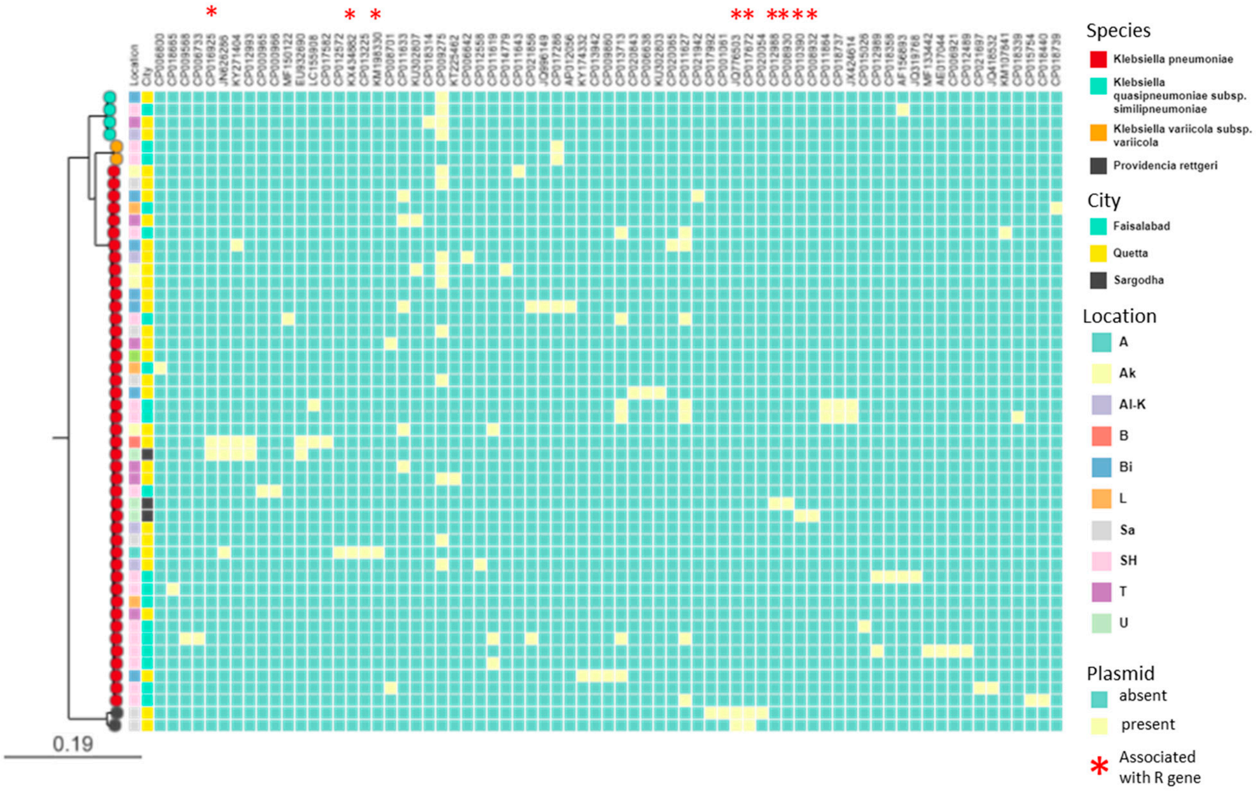

Supplementary Figure S4. Distribution of replicons.

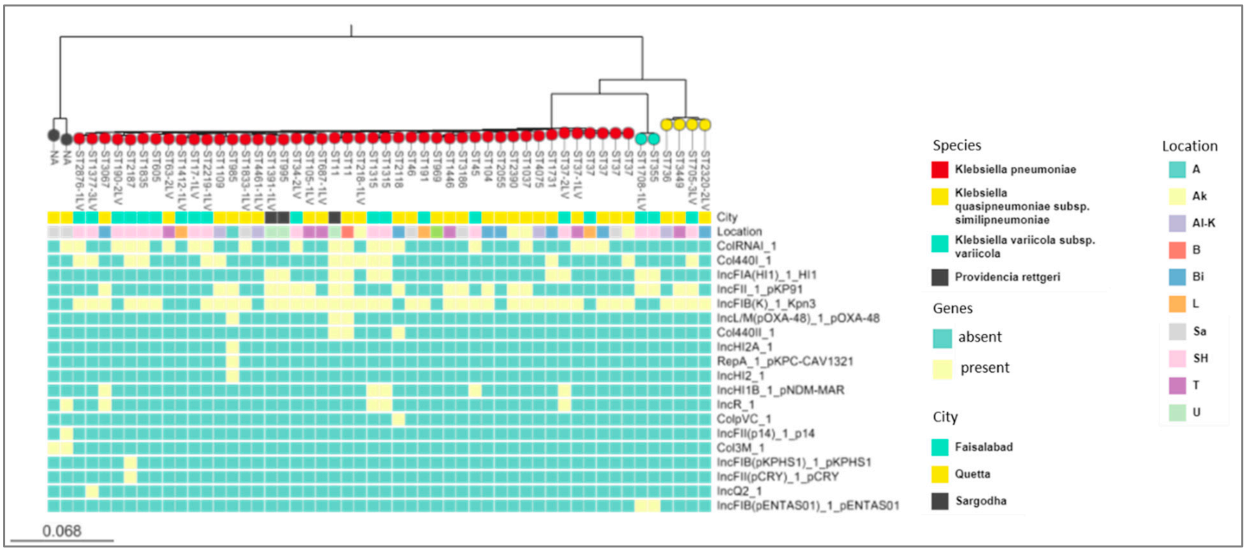

Supplement: Supplementary file 1 [file antibiotics-12-00539-s001.zip › antibiotics-2225727-supplementary.pdf]
